# Clinicopathologic features and biologic behavior of canine splenic nodules with stromal, histiocytic and lymphoid components

**DOI:** 10.3389/fvets.2022.962685

**Published:** 2022-08-12

**Authors:** Silvia Sabattini, Antonella Rigillo, Greta Foiani, Laura Marconato, Marta Vascellari, Alessandra Greco, Chiara Agnoli, Maurizio Annoni, Erica Melchiotti, Michela Campigli, Silvia Lucia Benali, Giuliano Bettini

**Affiliations:** ^1^Department of Veterinary Medical Sciences, Alma Mater Studiorum University of Bologna, Ozzano dell'Emilia, Bologna, Italy; ^2^Istituto Zooprofilattico Sperimentale delle Venezie, Padua, Italy; ^3^AniCura Clinica Veterinaria Tibaldi, Milan, Italy; ^4^Oncology Division, San Marco Veterinary Clinic and Laboratory, Padua, Italy; ^5^Laboratorio di Analisi Veterinarie MYLAV, Milan, Italy

**Keywords:** dog, spleen, complex nodular hyperplasia, splenic stromal sarcoma, histiocytic sarcoma, fibrohistiocytic nodule

## Abstract

The term fibrohistiocytic nodule has been discouraged in favor of specific pathologic entities, including complex nodular hyperplasia, splenic stromal sarcoma and histiocytic sarcoma. Nevertheless, the diagnosis of splenic lesions with mixed stromal, histiocytic and lymphoid components still remains a challenge due to lack of straightforward histologic criteria. Misestimation of the biologic behavior of these lesions may lead to detrimental consequences on the clinical management of patients. In this study, we retrospectively evaluated the clinicopathologic features and outcome of canine splenic nodular lesions with mixed components, to identify prognostic factors and histologic criteria of malignancy. Thirty-seven cases were included. Immunohistochemistry did not allow for further subclassification. Nine (24.3%) dogs died from disease-related causes after a median of 234 days (range, 48–1,247). One-, 2- and 3-year disease-specific survival rates were 80, 60, and 43%, respectively. When considering nodules with stromal cell atypia and at least one of mitotic count ≥9, presence of karyomegaly/multinucleated cells and lymphoid component <40%, half of these dogs died of disease-related causes with a median disease-specific survival time of 548 days (95% CI, 0-1216). In the remaining dogs, no disease-related death was reported (*P* < 0.001). Canine splenic nodular lesions with mixed stromal, histiocytic and lymphoid components and histologic criteria of malignancy may behave aggressively, leading to distant metastasis and death. In the absence of further criteria aiding their classification, and to better characterize their biologic behavior, we encourage the distinction of these complex splenic tumors from conventional sarcomas and histiocytic sarcomas.

## Introduction

The term fibrohistiocytic nodule (FHN) has been widely used in veterinary pathology up to 10 years ago to indicate canine splenic nodular lesions with heterogeneous cell components, including lymphocytes, histiocytes, hematopoietic precursors, fibroblasts and smooth muscle cells. FHNs comprised lesions with different biologic behavior that were interpreted as a continuum between hyperplastic and neoplastic lesions. Thus, they were classified into three grades based on the ratio between lymphoid and fibrohistiocytic components, with the increase of the latter corresponding to a greater biologic aggressiveness (1).

The diagnosis of FHN is now considered outdated, following a study which has reclassified immunohistochemically 31 lesions previously diagnosed as FHN, eventually identifying several different pathological entities, both neoplastic and non-neoplastic, including lymphoid nodular hyperplasia (LNH), complex nodular hyperplasia (CNH), marginal zone lymphoma, high grade B-cell lymphoma, splenic stromal sarcoma (SSS) and histiocytic sarcoma (HS) (2).

In routine histopathologic reporting, splenic nodular lesions with a clearly predominant stromal, histiocytic or lymphoid component are diagnosed as SSS, HS and LNH/follicular-derived lymphoma, respectively, with the latter showing a more benign biologic behavior (3). Nevertheless, a significant number of lesions is composed of almost equal proportions of stromal, histiocytic and lymphoplasmacytic elements. Such lesions could fall either into the category of benign splenic CNH or HS/SSS with associated lymphoid/histiocytic hyperplasia. However, straightforward histologic criteria to differentiate between these entities are lacking. The recent description of the category of “sarcoma arising from CNH” (4) further supports the hypothesis that these lesions may represent a continuum, and that nodules with intermediate characteristics between hyperplasia and malignant tumors may exist.

In addition, the biologic behavior of SSS is still largely uncharacterized, mostly due to its recent introduction and to the low diagnostic reproducibility. So, it is currently unknown whether chemotherapy is of benefit to dogs with SSS.

In this retrospective study, we evaluated the clinical presentation, histopathologic/immunophenotypic features and the biologic behavior of canine splenic nodules with mixed stromal, histiocytic and lymphoid components, in order to improve the understanding of their biologic behavior, identify prognostic factors and establish criteria for differentiating hyperplastic from malignant lesions.

## Materials and methods

### Study design

The histopathology databases of the Department of Veterinary Medical Sciences, University of Bologna (Italy), of the “Istituto Zooprofilattico Sperimentale delle Venezie,” Padua (Italy) of the “Clinica Veterinaria Privata e Laboratorio San Marco,” Veggiano (Italy) and of the “Laboratorio di Analisi Veterinarie MYLAV,” Milan (Italy), were retrospectively reviewed to identify canine splenic nodular lesions surgically removed between January 2011 and December 2021 and diagnosed as FHN, nodular hyperplasia, non-angiogenic sarcoma or HS.

The corresponding 4-μm-thick formalin-fixed and paraffin-embedded (FFPE) histologic samples, stained with hematoxylin and eosin (HE), were microscopically examined and the nodules composed of a mixed population of lymphoid, histiocytic and stromal cells were considered for inclusion.

To be eventually included, all the individual cell components had to be present and not to represent more than 70% of the cells in the nodule. Nodules composed by more than 70% of lymphoid, stromal or histiocytic elements were identified as nodular lymphoma/LNH, sarcomas and HS, respectively, and therefore excluded from the analysis. Similarly, we excluded nodules with a prominent hemorrhagic component impairing architectural and morphologic evaluations. Enrollment was accomplished by blind histologic review of the lesions by three of the authors (SS, AR and GF) and consensus on the achievement of the inclusion criteria, regardless of the former diagnosis.

Cases were ultimately included if data were available regarding demographics, treatment and follow-up. When available, additional information were recorded, including clinical signs at presentation, presence of a palpable abdominal mass by physical examination, presence of complete blood count (CBC), albumin to globulin (A/G) ratio or serum electrophoresis alterations, imaging tests performed, splenic nodule maximum diameter, nodule rupture, presence of additional splenic nodules, and presence of abdominal effusion,. Data were retrieved from referring clinicians.

Only blood tests performed no longer than 15 days before splenectomy were considered.

After inclusion, cases were histologically reviewed again, and the following histologic features were recorded: nodule demarcation, percentage and distribution of lymphocytes (assessed subjectively as percentage at low-medium magnification), spindle cells with nuclear atypia (e.g., anisokaryosis, variable nuclear shape, chromatin clumping and/or nucleolar prominence), presence of karyomegaly (defined as the presence of nuclei above 50 μm, identified at 10x magnification) and multinucleated cells (≥3 nuclei), mitotic count (MC, defined as the number of mitotic figures in a 2.37 mm^2^ area assessed outside lymphocyte-rich areas), presence of necrosis >10% and splenic veins thrombosis.

For a better characterization of the cell components, the following immunohistochemical antibodies were applied: vimentin, desmin, smooth-muscle actin (SMA) calponin, Iba1, CD18, CD163, E-cadherin, CD3, CD20. Additionally, Masson's trichrome stain was performed with a commercially available kit (Bio-Optica, Milan, Italy) to assess the amount of collagen within the nodules.

Histologic evaluation was performed by three of the authors (SS, AR and GF) and the final determinations were obtained by consensus.

### Immunohistochemistry

Automated immunohistochemistry (IHC) was performed on the Discovery ULTRA system (Roche, Ventana Medical Systems Inc., Tucson, AZ, USA) using primary antibodies anti-vimentin (Dako, Glostrup, Denmark), desmin (Dako), smooth muscle actin (SMA, Dako), calponin (Sigma-Aldrich, Merk Life Science S.r.l., Milano, Italy), Iba1 (FUJIFILM Wako Pure Chemical Corporation, Osaka, Japan), CD18 (Prof. Peter Moore-UC Davis School of Veterinary Medicine, CA, USA), CD163 (Abcam plc Discovery drive, Cambridge Biomedical Campus, Cambridge CB2 0AX, UK), E-Cadherin (BD Biosciences Transduction Laboratories, Franklin Lakes, NJ, USA), CD3 (Dako) and CD20 (Thermo Scientific, Waltham, MA, USA). Detailed information about the panel of primary antibody dilutions, source, retrieval, and detection systems is listed in Table 1.

**Table 1:** Panel of antibodies applied to 37 splenic nodular lesions with stromal, histiocytic, and lymphoid components.

**Antibody**	**Source**	**Clonality**	**Antigen retrieval**	**Antibody Incubation**	**Detection System**	**Positive control (canine)**
Vimentin	Mouse	Monoclonal (V9)	CC1^†^^*^ 95°C 24 min	1:100 20 min RT^¶^	Discovery OmniMap anti Mouse HRP^*^, ChromoMap DAB kit^*^	Skin
Desmin	Mouse	Monoclonal (D33)	CC1 95°C 32 min	1:100 20 min RT	Discovery OmniMap anti Mouse HRP,ChromoMap DAB kit	Intestine
Smooth-muscle actin (SMA)	Mouse	Monoclonal (1 A4)	CC1 95°C 32 min	1:200 32 min 37°C	Discovery OmniMap anti Mouse HRP, ChromoMap DAB kit	Intestine
Calponin	Mouse	Monoclonal (HCP)	CC2^§^^*^ 91°C 24 min	1:30000 40 min RT	Discovery OmniMap anti Mouse HRP, ChromoMap DAB kit	Mammary complex adenoma
Iba1	Rabbit	Polyclonal	CC1 95°C 24 min	1:500 32 min RT	Discovery anti Rabbit HQ+Anti HQ HRP,ChromoMap DAB kit	Cutaneous histiocytoma
CD18	Mouse	Monoclonal	CC1 95°C 16 min Protease2 36°C 4 min	1:100 32 min RT	Discovery OmniMap anti Mouse HRP, ChromoMap DAB kit	Lymph node
CD163	Rabbit	Monoclonal (EPR19512)	CC1 95°C 40 min	1:100 36 min 37°C	Discovery OmniMap anti Rabbit HRP, ChromoMap DAB kit	Lymph node
E-Cadherin	Mouse	Monoclonal (36/E)	CC1 95°C 32 min	1:150 32 min RT	Discovery OmniMap anti Mouse HRP.ChromoMap DAB kit	Intestine
CD3	Rabbit	Polyclonal	CC1 95°C 64 min	1:100 24 min 37°C	Discovery OmniMap anti Rabbit HRP,ChromoMap DAB kit	Lymph node
CD20	Rabbit	Polyclonal	CC1 95°C 32 min	1:200 1 h 37°C	Discovery OmniMap anti Rabbit HRP ChromoMap DAB kit	Lymph node

† *CC1, Discovery Cell Conditioning Solution 1 pH 8.4;*

¶ *RT, room temperature;*

§ *CC2, Discovery Cell Conditioning Solution 2 pH 6.0*.

* *Roche, Ventana Medical Systems Inc. (Tucson, AZ, USA)*.

Briefly, 3-μm-thick FFPE tissue sections were mounted onto superfrost plus slides, deparaffinized in aqueous-based detergent solution (Discovery Wash, Ventana) and underwent heat-induced antigen retrieval (Table 1). After detection, sections were counterstained with Mayer's hematoxylin (Hematoxylin II, Ventana), dehydrated and mounted with Eukitt (Kaltek, Padova, Italy). Positive controls from canine tissues and sections with omission of the primary antibodies (negative controls) were included in each run (Table 1).

### Statistical analysis

When appropriate, data sets were tested for normality by use of the Shapiro–Wilk test. Values were expressed as mean ± standard deviation in case of normal distribution, or as median with a range in case of non-normal distribution.

Survival time was defined as the number of days from the date of splenectomy to the date of death or to the date of the last visit if death did not occur. Dogs dead of causes related to the splenic disease were considered as events. Dogs were censored if alive at the end of the study or dead from other causes. Survival plots were generated according to the Kaplan-Meier product-limit method.

The influence of the evaluated variables on disease-related death was investigated with univariable and multivariable Cox proportional hazards model. Only covariates that tested significant at univariable analysis were included in the multivariable (adjusted) regression model. Clinical variables included age (median used as cut-off), sex, neutering status, body weight (median used as cut-off), presence of clinical signs at presentation, presence of a palpable abdominal mass by physical examination, presence of hematological alterations [anemia (Hct <37%), thrombocytopenia (platelets <160 × 103/μl), thrombocytosis (platelets >500 × 103/μl), abnormal neutrophils count (neutrophils <3,000/ μl or > 12,000/μl), decreased A/G ratio (<0.75)] (5), splenic nodule rupture, nodule diameter (median used as cut-off). Histologic variables included demarcation (well-vs poorly demarcated), percentage of lymphoid component (<40 vs. ≥40%) (1), prevalent distribution of the lymphoid component (follicular vs. diffuse), spindle cells with nuclear atypia, karyomegaly, multinucleated cells, MC (<9 vs. ≥9) (6), necrosis >10% and thrombosis.

The median disease-specific survival time (DSS) in dogs bearing lesions characterized by the presence of negative prognostic factors was compared with that of the remaining dogs by means of the log-rank test.

Data were analyzed with SPSS software (SPSS, Inc., IBM, Chicago, IL, USA). *P* ≤ 0.05 were considered significant.

## Results

### Dogs characteristics

Thirty-seven dogs were included in the analysis. There were 13 (35.1%) mixed breed dogs (4 small-sized, 8 medium-sized and 1 large-sized); among purebred dogs, the most represented breeds were Pug (*n* = 3; 12.5%), Beagle, Bernese Mountain dog, English bulldog and Maltese (*n* = 2 each; 8.3%). There were 22 (59.5%) males, of which 3 neutered and 15 (40.5%) females, of which 8 spayed. Median age was 10 years (range, 6–16). Median weight was 15.4 kg (range, 5.5–40.2).

Information about clinical signs was available for 33 (89.2%) dogs. Twenty-two of them (66.7%) were symptomatic at presentation. The most frequent clinical signs included asthenia (*n* = 15; 45.5%), dysorexia (*n* = 8; 24.2%), vomiting (*n* = 5; 15.2%) and weight loss (*n* = 3; 9.1%). In 6 of 31 (19.4%) dogs, a palpable mass was detected by physical examination. Eleven (33.3%) dogs were asymptomatic, and the splenic nodule was discovered accidentally during abdominal ultrasound (AUS) for unrelated causes (*n* = 9; 81.8%) or laparotomy (*n* = 2; 18.2%) for spaying and the removal of a gastric foreign body.

Information on hematologic abnormalities and serum chemistry was available for 21 (57%) dogs, while serum electrophoresis was available for 10 (27%) dogs. Observed CBC alterations included mild to moderate non regenerative anemia (*n* = 14; 67%), mild neutrophilia (*n* = 5; 24%), thrombocytopenia (*n* = 4; 19%), and thrombocytosis (*n* = 5; 24 %). Thirteen dogs (62%) had a decreased A/G ratio, and serum protein electrophoresis showed a polyclonal response in 10 (100%) dogs.

Beside AUS, which was performed in 35 (94.6%) dogs, 16 (43.2%) dogs underwent 3-view thoracic radiographs, 2 (5.4%) had 3-view thoracic radiographs and an echocardiogram performed, whereas 7 (18.9%) dogs underwent total body computed tomography scan. Two (5.4%) dogs underwent laparotomy only. None of the dogs had clinically apparent metastatic lesions at presentation.

Four (10.8%) dogs had low to moderate peritoneal effusion and one (2.7%) received a blood transfusion during splenectomy.

At macroscopic examination, 11 (29.7%) dogs had focal rupture of the splenic capsule. Median nodule diameter was 6 cm (range, 1–15). Six (16.2%) dogs had concurrent splenic nodules, which were diagnosed as nodular lymphoid hyperplasia (*n* = 4) or hematopoietic nodular hyperplasia (*n* = 2).

### Histologic and immunohistochemical examination

Included lesions had been formerly diagnosed as CNH (*n* = 18), SSS (*n* = 9), FHN (*n* = 4), undifferentiated tumor (*n* = 2), LNH (*n* = 1), HS (*n* = 1), SSS or HS (*n* = 1), and giant cell sarcoma (*n* = 1).

Seventeen (45.9%) nodules were well-demarcated and 20 (54.1%) were poorly demarcated.

All nodules were composed by a mixed proliferation of spindle-shaped and round cells.

**Figure 1:**
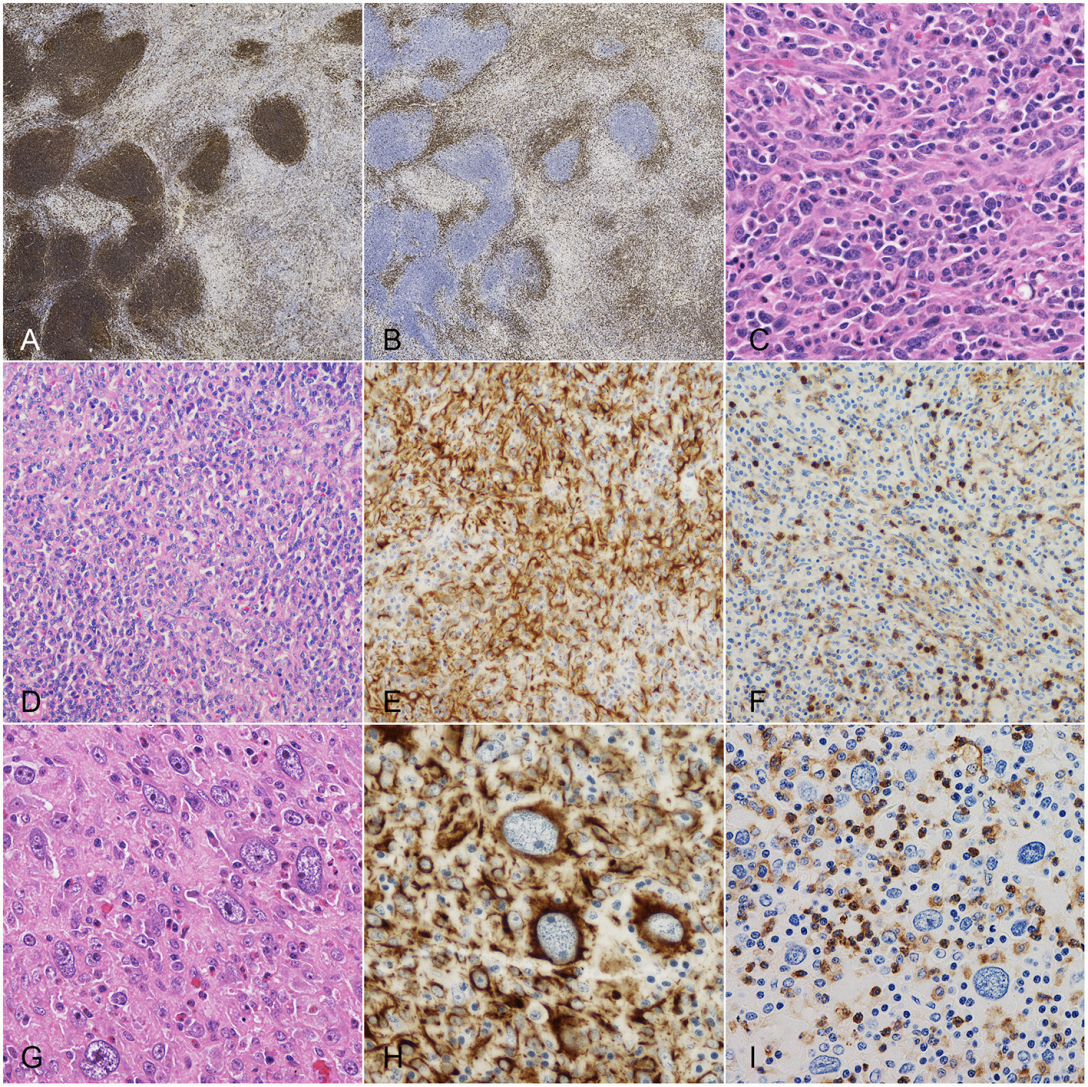
Dog, spleen. Main histologic characteristics observed in splenic nodules with mixed stromal, histiocytic and lymphoid components. **(A)** CD20 immunohistochemistry (IHC): positivity of lymphocytes arranged in follicles and of fewer diffusely-distributed lymphocytes. **(B)** CD3 IHC: positivity of the diffuse lymphoid infiltrate. **(C)** Hematoxylin and eosin (HE): spindle cells with nuclear atypia and plasma cell infiltrate. **(D)** HE: stromal, histiocytic and lymphoid components irregularly admixed. **(E)** Same field after desmin IHC, highlighting stromal cells. **(F)** Same field after CD18 IHC, highlighting histiocytic cells. **(G)** HE: cells displaying karyomegaly. **(H)** Desmin IHC: strong cytoplasmic positivity of karyomegalic cells. **(I)** CD18 IHC: diffuse negativity of karyomegalic cells.

Lymphocytes were predominately arranged in follicles in 18 (48.6%) cases and predominantly diffusely distributed in 19 (51.4%). Lymphocytes arranged in follicles were CD20-positive (B immunophenotype), whereas diffuse lymphoid infiltrates were primarily composed of CD3-positive (T immunophenotype) lymphocytes with smaller numbers of B-cells. According to Spangler and Kass classification, 8 (21.6%) nodules had a percentage of lymphoid cells below 40% and were therefore referable to grade 3 FHNs; the remaining 29 (78.4%) cases were referable to grade 2 FHNs (Figure 1).

Spindle cells were positive to both vimentin and desmin and negative to actin and calponin. Nuclear atypia was observed in 20 (54.1%) cases; karyomegaly and multinucleated cells were detected in 6 (16.2%) and 7 (18.9%) cases, respectively. MC ranged from 0 to 22 (median, 6). Ten (27.0%) lesions had a MC equal or higher than 9 (Figure 1).

Besides lymphocytes, large numbers of histiocytes/macrophages were irregularly admixed with spindle cells, as confirmed by CD18/Iba1 expression. On average, two-thirds of CD18-positive cells expressed CD163 (macrophage phenotype). Additionally, low to moderate numbers of E-cadherin-positive histiocytic cells (Langerhans phenotype) were scattered throughout the lesions. Nuclear atypia and multinucleated histiocytic cells were occasionally seen.

Overall, the majority of multinucleated cells seen in the nodules were negative to histiocytic markers and showed multifocal expression of desmin.

Finally, moderate to large numbers of plasma cells with or without scattered Mott cells and variable numbers of hemopoietic precursors and eosinophils were comprised among proliferating cells (Figure 1).

Eighteen (48.6%) nodules harbored a variable degree (>10%) of necrosis. Thrombosis was observed in 14 (37.8%) cases. Masson's trichrome stain evidenced a low amount of mature collagen in all lesions.

### Outcome

Following splenectomy, 3 (8.1%) dogs received adjuvant medical treatment, consisting of 6 cycles of single-agent doxorubicin (*n* = 2) or metronomic chlorambucil and thalidomide (*n* = 1).

**Figure 2:**
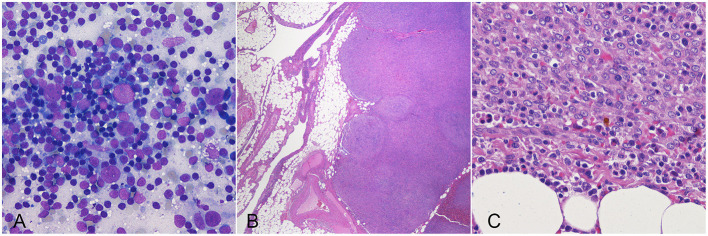
Dog, cytologic **(A)** and histologic **(B,C)** preparations of hepatic **(A)** and omental **(B,C)** metastasis from a splenic tumor. Lesions are composed by a pleocellular infiltrate of atypical stromal cells, histiocytes and lymphocytes, similar to the primary lesions.

Fifteen out of 37 (40.5%) dogs were still alive at data analysis closure, after a median follow-up of 618 days (range, 167–2,312). Thirteen (35.1%) dogs were dead from disease-unrelated causes after a median of 588 days (range, 135–1,799). Nine (24.3%) dogs died from disease-related causes after a median of 234 days (range, 48–1,247). One- 2- and 3-year disease-specific survival rates were 80, 60, and 43%, respectively. Dogs reported dead from disease-related causes were diagnosed with multiple nodular lesions in the liver (*n* = 4; 44.4%), peritoneum (*n* = 3; 33.3%); liver and peritoneum (*n* = 1; 11.1%) and lungs (*n* = 1; 11.1%) in the absence of other malignancies. Metastases were confirmed by cytology or histology in three cases and were composed of a pleocellular infiltrate of atypical stromal cells, histiocytes and lymphocytes, consistent with that of the primary lesion (Figure 2).

On univariable analysis, factors significantly associated with an increased risk of disease-related death included nodule diameter >6 cm, lymphoid component <40%, spindle cells with nuclear atypia, karyomegaly, multinucleated cells, MC ≥9 and thrombosis (Table 2). Additionally, all dogs dying from disease-related causes had nodules with a diffuse lymphocyte distribution and necrotic areas.

**Table 2:** Univariable Cox regression analysis of variables potentially associated with an increased risk of disease-related death in 37 dogs with mixed stromal, histiocytic and lymphoid splenic nodular lesions.

**Variable**	**Hazard ratio**	**95% CI**	***P*** **-value**
Age ≥ 10 years	4.29	0.84–22.01	0.081
Male sex	1.80	0.42–7.73	0.428
Neutering	0.25	0.03–2.04	0.196
Body weight ≥ 15 kg	0.80	0.21–3.00	0.743
Clinical signs at presentation	3.72	0.45–30.57	0.222
Palpable abdominal mass	1.95	0.38–10.13	0.426
Anemia	2.54	0.27–23.58	0.412
Neutrophilia	4.29	0.81–22.69	0.087
Thrombocytopenia	5.16	0.72–37.22	0.103
Thrombocytosis	0.70	0.70–6.96	0.760
Decreased A/G ratio	0.64	0.10–4.08	0.637
Splenic nodule rupture	1.91	0.42–8.78	0.406
Nodule diameter ≥ 6 cm	8.45	1.03–69.67	0.047^*^
Poor demarcation of nodule	1.99	0.49–7.99	0.334
Lymphoid component <40%	4.25	1.13–15.93	0.032^*^
Diffuse distribution of lymphoid component	73.32	0.34–1586.97	0.117
Spindle cells with nuclear atypia	10.64	1.32–85.68	0.026^*^
Karyomegaly	4.43	1.02–19.20	0.047^*^
Multinucleated cells	17.02	3.16–91.76	0.001^*^
MC ≥ 9	10.93	2.26–52.98	0.003^*^
Necrosis >10%	86.22	0.38–197.78	0.108
Thrombosis	5.57	1.15–26.92	0.032^*^

* *Significant at the 5% level. CI, confidence interval; A/G, albumin to globulin; MC, mitotic count*.

**Figure 3:**
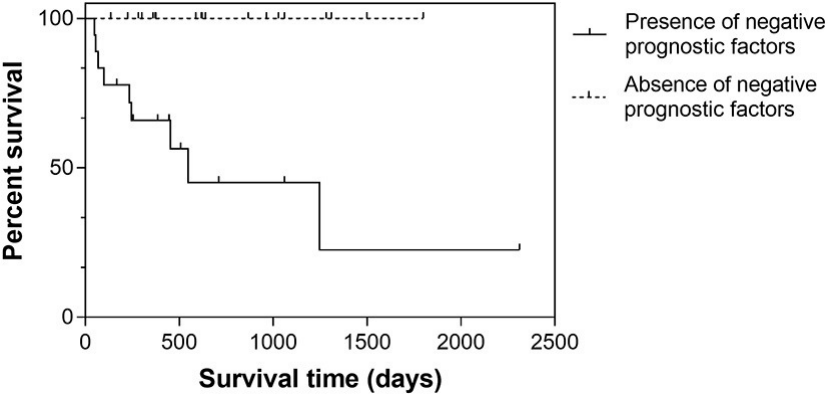
Kaplan-Meier survival curves in 37 dogs with splenic nodular lesions composed of a mixed stromal, histiocytic and lymphoid population. Cases were stratified according to the presence or absence of negative histologic prognostic factors (i.e., stromal cell atypia and at least one of MC ≥9, presence of karyomegaly/multinucleated cells, and lymphoid component <40%).

When considering nodules with stromal cell atypia and at least one of MC ≥9, presence of karyomegaly/multinucleated cells, and lymphoid component <40%, half of these dogs died of disease-related causes with a median disease-specific survival time of 548 days (95% CI, 0–1,216). In the remaining dogs, no disease-related death was observed (*P* < 0.001; Figure 3).

Of the three dogs receiving adjuvant chemotherapy, two had lesions with negative prognostic factors and were still alive at the end of the study after 167 and 255 days. The remaining dog died for disease-unrelated causes (hepatic cholangiocarcinoma diagnosed cytologically) after 360 days.

## Discussion

This study is focused on canine splenic nodules with mixed stromal, histiocytic and lymphoid components: a long-discussed entity with still many unanswered questions regarding histologic classification, biologic behavior and indications for adjuvant treatment.

Since the use of the umbrella term FHN has been discouraged, veterinary pathologists are expected to make a specific diagnosis of these lesions, and the choice must fall between a hyperplastic disease and a malignant neoplasm. This assessment, even with the support of IHC, is still subjective, especially in cases lacking a clear predominance of a single cell population. The incorrect evaluation of the biologic behavior of these lesions may lead to detrimental consequences on the clinical management of patients, such as underestimation of the risk or overtreatment, depending on the presumptive and definitive diagnosis.

The reclassification study by Moore et al. was performed on cases with a former diagnosis of FHN made by different pathologists with no further revision. This might explain the inclusion of lesions that deviate from the classic morphologic appearance of FHN, such as marginal zone lymphoma and high-grade B-cell lymphoma (2). In the present study, splenic nodular lesions with different diagnoses were histologically reviewed and enrollment was based on histologic appearance: only cases with a mixed population of stromal cells, histiocytes and lymphocytes were ultimately included. IHC was performed on all cases, and confirmed the coexistence of the three cellular components, demonstrating a poor variability in the immunoreactivity pattern among cases and preventing further classification.

Nevertheless, we observed a variable biologic behavior among the included cases, supporting the previous hypothesis that a *continuum* may exist between hyperplastic and neoplastic lesions (1, 4). In particular, distant metastases and disease-related death were observed in 9 dogs, proving that these lesions may progress like a malignant neoplastic disease. Yet, their histologic appearance is largely different from that of conventional sarcomas or HS, which are not usually associated with prominent hyperplastic proliferation of other cell types. At least in some cases, such pleocellular appearance is also maintained in metastatic lesions, as observed in this study and reported by Spangler and Kass (1). Most importantly, the criteria of malignancy that should shift a diagnosis of CNH to a diagnosis of SSS or HS are poorly defined and, presently, it is unknown whether their biologic behavior is similar to that of conventional sarcomas or HS.

A diagnosis of HS based solely on the presence of scattered multinucleated cells and on multifocal immunohistochemical positivity to histiocytic markers might not be straightforward and have relevant clinical consequences. In the present study, most multinucleated cells were negative to histiocytic markers and showed multifocal expression of desmin, suggesting a mesenchymal origin. Additionally, the median survival time of dogs that died of disease-related causes (234 days with no adjuvant medical treatment) is quite divergent from the survival times usually reported for canine HS, a highly aggressive disease requiring specific chemotherapeutic treatment. Recently, a localized form of splenic HS with longer survival (median, 349–427 days) has been reported; possibly the inclusion of these mixed lesions might have contributed to increase the survival times of dogs in those studies (7, 8).

In the reclassification study by Moore et al., a very short survival time was reported for dogs with HS (median, 74 days, range 2–146 days), whereas a longer survival was observed for CNH and SSS (2). Nevertheless, notably, no better outcome was observed in dogs with CNH compared with SSS, with a longer median survival time reported in the latter (387 vs. 488 days, respectively), suggesting the need of clinical prospective studies to better understand the behavior of these lesions (2).

For the above reasons, we encourage the distinction of these mixed splenic lesions from conventional sarcomas/HS. In the future this may allow for a better comprehension of their specific biologic behavior and optimal clinical approach.

Further, we focused on histomorphologic criteria associated with a negative outcome, in the attempt to identify the patients requiring additional medical intervention following splenectomy. These criteria are mostly in agreement with the grading system proposed in the original paper by Spangler and Kass and include stromal cell atypia, high mitotic activity, presence of karyomegaly/multinucleated cells, necrosis, and a lymphoid component <40%, mainly composed by diffusely distributed T-cells. Moreover, nodules larger than 6 cm were associated with aggressive behavior.

The immunohistochemical positivity of the stromal component for desmin suggests a myofibroblastic origin. Interestingly, the histologic features of the lesions included in this study are strikingly similar to those of human inflammatory myofibroblastic tumor (IMT), a rare intermediate-grade mesenchymal tumor documented in children and young adults, characterized by proliferation of fibroblasts and myofibroblasts, accompanied by chronic inflammatory infiltration of lymphocytes, plasma cells, eosinophils and histiocytes (9). The etiopathologic mechanism of IMT origin remains not fully understood. However, researchers suppose various antigens might play an important role in abnormal inflammatory response as an immunological trigger. Virus-induced trauma, surgery, autoimmune etiology, inflammation, infection, and abnormal responses to long-standing exogenous stimuli may result in tumorigenesis (9, 10). IMT has been rarely reported in dogs in various anatomic location, including skin, pancreas, urinary bladder and retrobulbar region, although the immunohistochemical expression of muscular markers was different from that reported in the current study (11–13).

A further relevant finding, although not significantly correlated with biologic behavior, was the frequent occurrence of hematologic abnormalities. These mainly included mild to moderate non-regenerative anemia, decreased A/G ratio, and the electrophoretic evidence of polyclonal hypergammaglobulinemia. These findings are generally observed in inflammatory processes, including infectious or autoimmune disease. Notably, polyclonal hypergammaglobulinemia has never been reported before in canine splenic nodular neoplastic/hyperplastic lesions and might correlate with the histologic finding of splenic inflammatory cell infiltration, including lymphocytes and plasma cells. A better understanding of the course of these alterations following splenectomy could be the first step to recognize their relevance and pathogenesis.

The main limits of this study reside in the low number of cases, owing to the relative infrequency of these lesions and the strict histologic criteria applied for inclusion. The retrospective study design resulted in poorly standardized staging work-ups and patient management. Additionally, partly due to the variability of the original histopathologic diagnoses, most patients were not regularly followed-up and suspected metastatic lesions were not always confirmed histologically or cytologically. The utility of adjuvant medical therapy could not be determined, as only 3 dogs were treated with chemotherapy and follow-up was too short at data analysis closure to draw ultimate conclusions. Finally, the wide immunohistochemical panel applied in this study lacked the presence of podoplanin, that was recently advocated as a useful diagnostic marker for SSS (4). Regrettably, a preliminary analysis did not return satisfying results on podoplanin immunoreactivity (data not shown) and the antibody was not included in the study.

In conclusion, a gray zone still remains in the classification of splenic nodular disease, consisting of lesions with histologic criteria of malignancy and pleocellular stromal, histiocytic and lymphoid components. While not being attributable to one specific origin based on morphology or immunohistochemistry, nearly half of these lesions may have an aggressive biologic behavior, leading to distant metastasis and death. In the absence of further criteria aiding their classification, we propose to use the term “complex splenic tumor” to refer to splenic nodular lesions with stromal, histiocytic and lymphoid components not reaching individually 70% of the nodule and with criteria of malignancy (i.e., stromal cell atypia and at least one of MC ≥9, presence of karyomegaly/multinucleated cells, lymphoid component <40%, necrosis, nodule diameter >6 cm). For those not showing criteria of malignancy, the term of complex nodular hyperplasia could be maintained. This differentiation might allow for a more thorough comprehension of their specific biologic behavior and optimal treatment strategies.

## Data availability statement

The raw data supporting the conclusions of this article will be made available by the authors, without undue reservation.

## Ethics statement

Ethical review and approval was not required for the animal study because this study was purely observational, only describing routine patient care. Written informed consent was obtained from the owners for the participation of their animals in this study.

## Author contributions

SS and AR conceived the idea and planned the study. AR, GF, LM, AG, MA, CA, MC, and SB collected the data. SS, AR, and GF performed the histological and immunohistochemical evaluations. SS performed the statistical analysis and the interpretation of data. SS, AR, EM, and AG drafted the manuscript and prepared the tables and the figures. LM, CA, MV, EM, and GB revised the manuscript. All authors discussed the results and approved the final version of the manuscript.

## Conflict of interest

The authors declare that the research was conducted in the absence of any commercial or financial relationships that could be construed as a potential conflict of interest.

## Publisher's note

All claims expressed in this article are solely those of the authors and do not necessarily represent those of their affiliated organizations, or those of the publisher, the editors and the reviewers. Any product that may be evaluated in this article, or claim that may be made by its manufacturer, is not guaranteed or endorsed by the publisher.
